# Chronic pyelonephritis presenting as a renal sinus tumor with retroperitoneal extension: a case report

**DOI:** 10.4076/1752-1947-3-9054

**Published:** 2009-09-15

**Authors:** Georgios I Papadopoulos, Ioannis G Mountanos, Stylianos I Manolakakis, Georgios Chrysanthakopoulos, Eugenia Papaliodi, Antonios D Farmakis

**Affiliations:** 1 Department of Urology, Athens General Hospital "G. Gennimatas", 154 Mesogeion av, 11527, Athens, Greece; 2 Evroiatriki Protypo Diagnostiko Messinias, Artemidos str, 24100, Kalamata, Greece; 3 Department of Pathology, Athens General Hospital "G. Gennimatas", 154 Mesogeion av, 11527, Athens, Greece

## Abstract

**Introduction:**

Chronic pyelonephritis is associated with progressive renal scarring and occurs, most of the time, in patients with major anatomical anomalies, including urinary tract obstruction, calculi, renal dysplasia or vesicoureteric reflux. We report the computed tomography imaging findings of a patient with chronic pyelonephritis appearing as a renal sinus mass. To our knowledge, it is the first time that such a case has been published in the literature.

**Case presentation:**

We present a case of a 68-year-old woman who underwent a computed tomography scan of the abdomen in the work-up for recently diagnosed hypertension. A non-enhancing left renal sinus mass was detected extending to the para-aortic space. The initial diagnosis was that of a tumor of the collecting system. Nephro-ureterectomy was performed and the pathology results revealed changes of chronic pyelonephritis.

**Conclusion:**

Chronic pyelonephritis presenting as a renal sinus mass is reported for the first time in the literature. This may lead to the conclusion that diagnostic ureteropyeloscopy and biopsy should be performed prior to radical surgery for possible upper tract urothelial tumors.

## Introduction

Chronic pyelonephritis represents a renal injury induced by recurrent or persistent renal infection, associated with progressive renal scarring. This may also lead to end-stage renal disease [1]. Imaging usually reveals the presence of renal obstruction, calculi, renal dysplasia or scarring [2,3]. The case we report here is unusual because of the absence of any of these typical findings.

## Case presentation

A 68-year-old woman was referred for a computed tomography (CT) scan of the abdomen because of recently diagnosed drug-resistant hypertension. Her past medical history was unremarkable, and her physical examination and general biochemical assays were normal. A spiral CT scan was performed with unenhanced and contrast-enhanced images after bolus intravenous injection of 50 ml of non-ionic contrast medium, in the nephrographic and the excretory phases. Axial 8 mm- and 5 mm- thick images were reconstructed from the original spiral data set.

**Figure 1:**
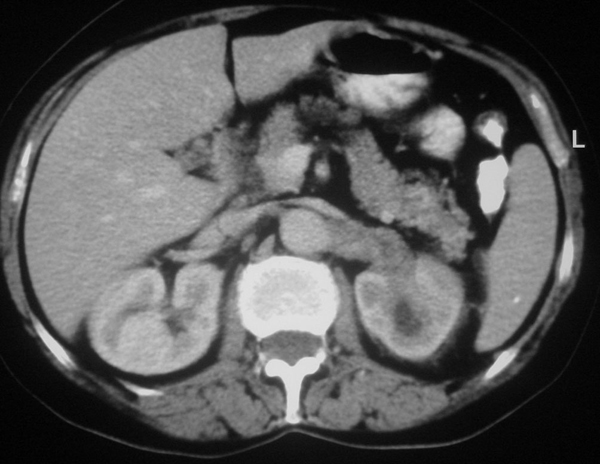
**Axial post-contrast computed tomography scan, with normal cortical enhancement of the left kidney and the non-enhancing mass extending to the left para-aortic space along its vascular pedicle**.

**Figure 2:**
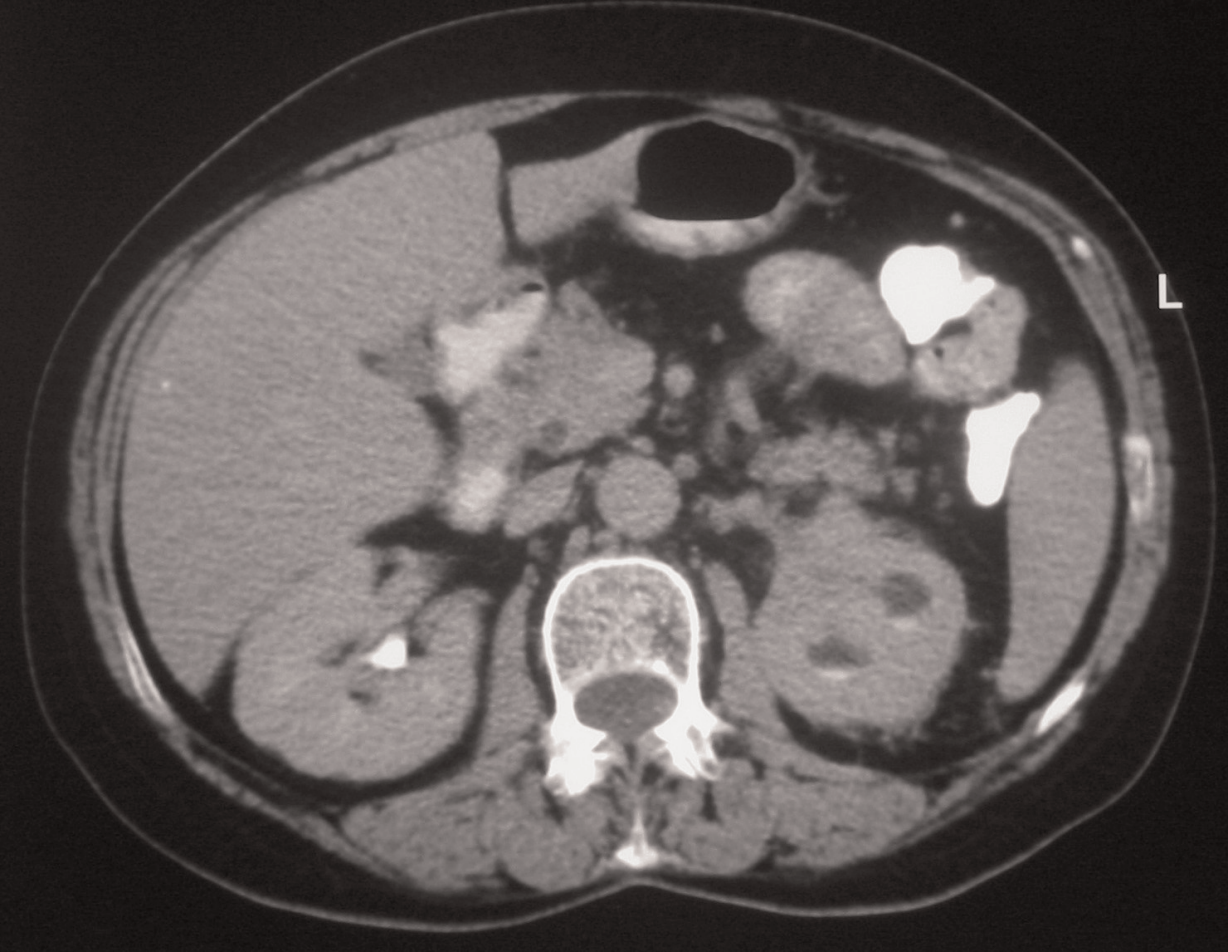
**Axial computed tomography scan revealing faint excretion of contrast medium in the lower pole calyces**.

The CT scan revealed an obstructing left renal sinus mass, practically non-enhancing, that extended along the vascular pedicle of the kidney to the left para-aortic area. Cortical thickness was within normal limits. There was no evidence of scarring. The nephrogram of the kidney was also normal compared to the opposite side (Figure 1), there were no stones in the pelvis or ureter, and faint excretion of contrast medium was evident in the lower pole calyces (Figure 2).

**Figure 3:**
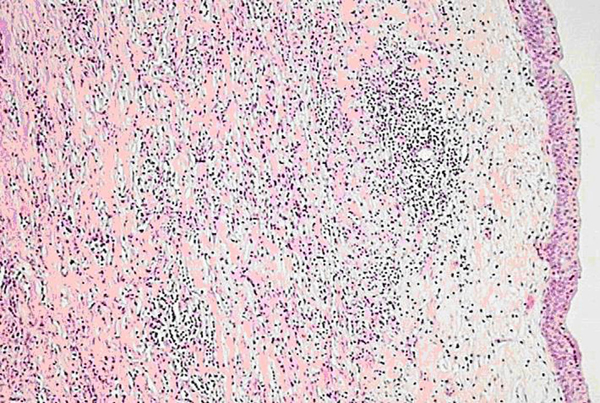
**Chronic inflammation of the renal pelvis (hematoxylin-eosin stain)**.

**Figure 4:**
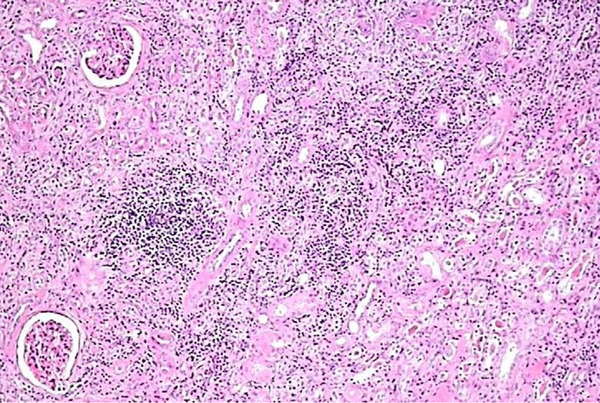
**Chronic interstitial infiltration of the kidney (hematoxylin-eosin stain)**.

A tentative diagnosis of a renal sinus tumor was made, and the patient underwent nephro-ureterectomy. Histology revealed findings of chronic pyelonephritis, namely hyalinized glomeruli, atrophy and focally cystic dilation of a few tubules, as well as chronic inflammatory infiltrate of the interstitial tissue. The inflammatory infiltrate extended to the pelvis with focal edema (Figures 3 and 4). Chronic inflammatory infiltrate was also noticed at the hilum around the ureter and the large vessels.

## Discussion

Chronic pyelonephritis is a renal injury induced by recurrent or persistent renal infection. It occurs, in most cases, in patients with major anatomic abnormalities, including urinary tract obstruction, renal dysplasia or, most commonly, vesicoureteric reflux in children [1,4,5]. Typical imaging findings include a small kidney with abnormal contour, scarring, as well as calyceal dilatation with or without the presence of stones [2,3]. We report an unusual case of a chronic inflammatory pseudotumor in the form of a renal sinus mass with para-aortic extension without the presence of either stone disease or renal contour abnormalities, and which was initially misinterpreted as a renal sinus tumor.

Differential diagnosis of renal masses of infectious origin that can be misinterpreted as tumors, includes focal xanthogranulomatous pyelonephritis and focal renal malakoplakia; conditions that both have distinct imaging and pathology features [6,7].

## Conclusions

Here, we present the CT imaging findings of a patient with a chronic inflammatory pseudotumor presenting as a renal sinus mass. To our knowledge this is the first such report in the literature. The conclusion to be drawn from this case might be that diagnostic ureteropyeloscopy and biopsy should be performed before any radical surgery for upper tract urothelial tumors.

## Consent

Written informed consent was obtained from the patient for publication of this case report and any accompanying images. A copy of this is available for review by the Editor-in-Chief of this journal.

## Competing interests

The authors declare that they have no competing interests.

## Authors' contributions

GIP and GC collected the data, conducted the literature review and wrote the manuscript. GIP was the main author. IGM and SIM were the radiologists who diagnosed the renal mass and prepared the CT pictures for this paper. EP carried out the pathology examination. ADF was the main surgeon who performed the nephro-ureterectomy. All authors read and approved the final manuscript.

## References

[B1] Roberts JA Mechanisms of renal damage in chronic pyelonephritis (reflux nephropathy). Review Curr Top Pathol 1995 88 265 287 7614849 10.1007/978-3-642-79517-6_9

[B2] Kawashima A Sandler CM Goldman SM Raval BK Fishman EK CT of renal inflammatory disease Radiographics 1997 17 851 866 9225387 10.1148/radiographics.17.4.9225387

[B3] Kawashima A LeRoy AJ Radiologic evaluation of patients with renal infections Infect Dis Clin North Am 2003 17 433 456 10.1016/S0891-5520(03)00007-2 12848478

[B4] Polito C La Manna A Rambaldi PF Valentini N Marte A Lama G Long-term evolution of renal damage associated with unilateral vesicoureteral reflux J Urol 2007 178 1043 1047 10.1016/j.juro.2007.05.061 17632145

[B5] Dunmore FR Chronic atrophic pyelonephritis in children. Review Adv Nurse Pract 2004 12 49 50 15559567

[B6] Shah M Haaga JR Focal xanthogranulomatous pyelonephritis simulatig a renal tumor: CT characteristics J Comput Assist Tomogr 1989 13 712 713 10.1097/00004728-198907000-00035 2745796

[B7] Mollier S Descotes JL Pasquier D Coquillat P Michel A Dalsoglio S Rambeaud JJ Pseudoneoplastic xanthogranulomatous pyelonephritis. A typical clinical presentation but unusual diagnosis and treatement Eur Urol 1995 27 170 173 7744162

